# Heat to Hydrogen by RED—Reviewing Membranes and Salts for the RED Heat Engine Concept

**DOI:** 10.3390/membranes12010048

**Published:** 2021-12-30

**Authors:** Pauline Zimmermann, Simon Birger Byremo Solberg, Önder Tekinalp, Jacob Joseph Lamb, Øivind Wilhelmsen, Liyuan Deng, Odne Stokke Burheim

**Affiliations:** 1 Department of Energy and Process Engineering, Norwegian University of Science and Technology (NTNU), NO-7491 Trondheim, Norway; pauline.zimmermann@ntnu.no (P.Z.); simon.b.b.solberg@ntnu.no (S.B.B.S.); jacob.j.lamb@ntnu.no (J.J.L.); 2 Department of Chemical Engineering, Norwegian University of Science and Technology (NTNU), NO-7491 Trondheim, Norway; onder.tekinalp@ntnu.no (Ö.T.); liyuan.deng@ntnu.no (L.D.); 3 Department of Chemistry, Norwegian University of Science and Technology (NTNU), NO-7491 Trondheim, Norway; oivind.wilhelmsen@ntnu.no

**Keywords:** hydrogen production, reverse electrodialysis, waste heat utilization, ion-exchange membranes, closed loop, heat engine, power production cycle

## Abstract

The Reverse electrodialysis heat engine (REDHE) combines a reverse electrodialysis stack for power generation with a thermal regeneration unit to restore the concentration difference of the salt solutions. Current approaches for converting low-temperature waste heat to electricity with REDHE have not yielded conversion efficiencies and profits that would allow for the industrialization of the technology. This review explores the concept of Heat-to-Hydrogen with REDHEs and maps crucial developments toward industrialization. We discuss current advances in membrane development that are vital for the breakthrough of the RED Heat Engine. In addition, the choice of salt is a crucial factor that has not received enough attention in the field. Based on ion properties relevant for both the transport through IEMs and the feasibility for regeneration, we pinpoint the most promising salts for use in REDHE, which we find to be KNO_3_, LiNO_3_, LiBr and LiCl. To further validate these results and compare the system performance with different salts, there is a demand for a comprehensive thermodynamic model of the REDHE that considers all its units. Guided by such a model, experimental studies can be designed to utilize the most favorable process conditions (e.g., salt solutions).

## 1. Introduction

A significant and rapidly growing share of renewable energy is produced intermittently, causing a mismatch between supply and demand. Known sources for renewable energy include solar, wind, geothermal, biomass, hydro, tidal, wave, and marine current energy [1]. In the quest for sustainable solutions for energy storage, Salinity Gradient Energy (SGE) has gained increasing attention in recent years [1,2,3]. The mixing of fresh water and seawater (e.g., as observed at river outlets flowing into ocean water) produces enormous amounts of entropy. The chemical potential difference between the two streams can be a source of sustainable energy by controlled mixing through a membrane and cyclic charging and discharging. The term blue energy has been coined for technologies exploiting salinity gradients for energy production [4]. The theoretical potential of salinity gradient power for the discharge of all river water worldwide into the sea was initially estimated to be 1.4 to 2.6 TW in the 1970s [5,6]. More recent studies suggest a global potential for salinity gradient power of slightly below 1 TW when factoring in technical and physical limitations [7]. In comparison, the global electricity demand in 2019 was around 2.85 TW [8]. The main benefits of SGE are no emission of CO_2_ and no consumption of the salts in the process, being the source of energy. Furthermore, SGE does not give time-discontinuities in power production as many other renewable energy sources and is suitable for continuous power production [1].

**Figure 1:**
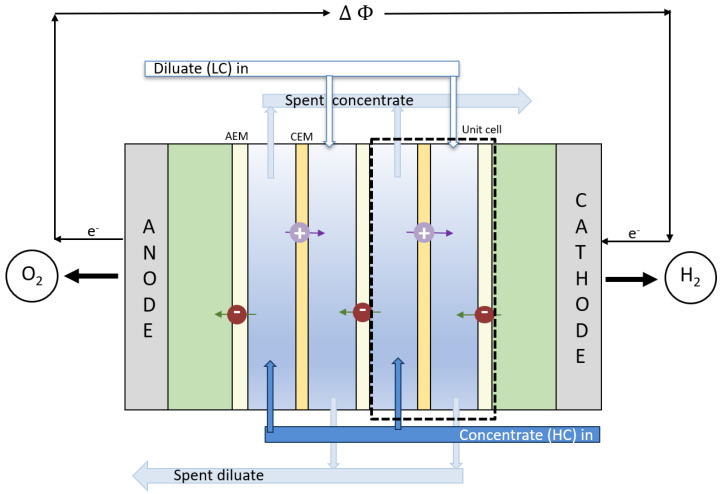
Illustration of a RED stack to be used as the power unit in the REDHE. The stack comprises *N* number of unit cells with alternating AEMs and CEMs giving an electric potential, Δϕ, which drives oxygen and hydrogen evolution at the anode and cathode, respectively. As a result, cations and anions migrate in opposite directions.

The concept of converting salinity gradients to energy by mixing fresh and saltwater was first introduced by Pattle in the 1950s [9]. Since then, various groups have studied the potential for power generation by SGE using different technologies [10,11,12]. At present, pressure retarded osmosis (PRO) and RED are the most promising technologies for exploiting salinity differences in naturally occurring waters [1,2,13,14]. In RED, controlled mixing is achieved by separating alternate layers of freshwater and saltwater with alternating cation and anion exchange membranes (AEMs/CEMs). The result is a net flux of ions through the stack. Electrodes on each stack end convert the ionic current to electric current conducted through an external circuit [15,16]. A schematic sketch is given in Figure 1.

In 2010, the EU-funded project *REAPower* was launched, aiming to develop a prototype for RED power generation from natural saturated brines from salt ponds (more concentrated than seawater) and brackish water (instead of freshwater) [11]. Using saturated brines as high-concentration (HC) and brackish water as low-concentration (LC) feed streams reduces the dilute compartment’s electrical resistance, increasing the achievable power output compared to seawater and freshwater feed streams [12,17,18,19,20]. A prototype with a total membrane area of almost 50 $m^{2}$ and a power output up to 40 W was commissioned in 2014 in Trapani, Italy [21].

**Figure 2:**
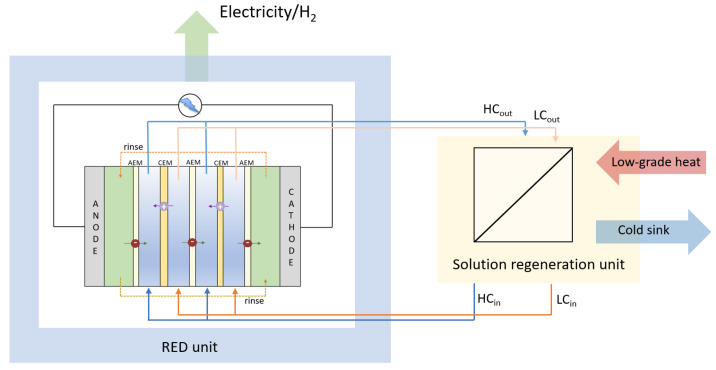
Schematic drawing of a RED Heat Engine, modified from [23]. Low-grade heat is added to the system while withdrawing electricity. High and low-concentration solutions are fed to the RED stack, mixed across AEMs and CEMs to convert their chemical potential into electricity, and then recycled to the solution regeneration unit to restore the initial chemical potential.

By coupling a SGE unit with a regeneration unit that restores the initial concentrations of the HC and LC feed stream, a closed-loop SGE engine is obtained. The concept is shown in Figure 2, with a RED stack as a power unit and a thermal regeneration unit. An essential benefit of the RED Heat Engine and Salinity Gradient Energy Storage is the possibility of closed systems with solid control of fouling challenges [22]. Using low-grade waste heat enhances the overall energy efficiency of the production cycles and the exploitation of an energy source that is readily available [23]. Furthermore, while a conventional RED stack for power production depends on the availability of both dilute and concentrate solutions at the same site, the REDHE is run on a finite recirculated amount of salt solution. This allows for much more flexibility in the system’s location and liberates it from the demand of working with readily available solutions found in the environment (e.g., seawater and river water). Consequently, the working solution can be selected by primarily optimizing the energy production, which is substantially determined by the properties of the salt solution, such as concentration, temperature, and presence of multivalent ions [7]. By using synthesized salt solutions with optimal characteristics for RED, problems as membrane fouling, or the necessity for pre-treatments to mitigate membrane-fouling, can be avoided leading to cost and energy savings. However, the concept of a REDHE as a closed-loop system requires regeneration of the working fluids (i.e., restoring the initial concentration gradient of the LC and HC stream after passing through the power unit).

Loeb patented the method and apparatus for a heat engine using PRO in 1975 [24] and for a heat engine using RED with a thermal regeneration unit in 1979 [25]. The scientific and engineering efforts for SGE utilization historically intensified after oil shortages or an increased societal awareness of the need for waste renewable energy sources [26,27].

In terms of industrial waste heat in the USA, the majority is released at low temperatures. Over 800 TWh of the waste heat is released at temperatures between 50 and 100 ${}^{\circ }$C [28], and in Germany, around 45 TWh of economically recoverable waste heat from big industrial plants is released at temperatures below 140 ${}^{\circ }$C [29]. Nevertheless, the existing technologies for waste heat utilization concentrate on high and medium temperature ranges (around 100 to 650 ${}^{\circ }$C) due to limitations in low-temperature waste heat recovery [30]. Established technologies for converting waste heat to power rely on producing mechanical energy through turbines and further conversion to electricity by a generator. An example is the steam Rankine cycle [31]. A range of novel technologies has been developed to convert waste heat directly to electric energy. However, few of these technologies operate with waste heat at very low temperatures (i.e., below 60 to 90 ${}^{\circ }$C). To date, none of the tested approaches were suitable for converting low-temperature waste heat to electricity at efficiencies and costs feasible for industrial application [30]. Instead of harvesting electricity, the electrical current generation from RED could be used to produce hydrogen gas, thereby presenting a method for renewable hydrogen gas production. Hydrogen production through water electrolysis is already broadly discussed as a promising energy storage technology [32,33,34,35,36]. More recently, Hatzell et al. [37] assessed the potential of hydrogen production in a closed-loop ammonium bicarbonate RED system and compared it to electrical power generation in the same system. It is reported that by recovering hydrogen gas from the RED system, the produced energy can be 1.5 times higher (118 $\mathrm{Wh}\;m^{-3}$), compared to directly withdrawing electricity. Both electricity and hydrogen production have large markets; however, there are limited technologies available to date for direct renewable hydrogen production. Of the yearly global hydrogen production of around 500 billion $m^{3}$, roughly 96% is produced using non-renewable fossil fuels, in particular through steam reforming of methane [33,38,39,40,41,42]. In that sense, hydrogen production through REDHEs could potentially fill a market niche, making the technology competitive with other renewables in the energy sector. Moreover, the produced hydrogen gas can be considered carbon-neutral, and its production does not require grid-based energy [37].

Former studies on REDHE focus on process design and optimization [3,43], as well as the role of the electrolyte in REDHE for converting waste heat to electricity [23,44]. While several of the studies have pointed out that membrane optimization is crucial for maximizing the power density of the REDHE, little attention has been given to how to achieve superior membrane properties. We, for the first time, give an extensive overview on feasible membrane properties for use in REDHE, reviewing studies on RED performed using commercial membranes and tailor-made membranes. We analyze the pertinent relations between membrane properties and process performance for both the solution regeneration and the reverse electrodialysis stack to map crucial directions for membrane development. We further introduce the alternative of producing hydrogen for energy storage rather than electricity, and thereby expand the green hydrogen market. Therefore, this review aims to: (1) give an overview of the state-of-the-art for the REDHE technology, its promises, and limitations, especially with regards to hydrogen production from waste heat; (2) compare different approaches for thermal solution regeneration; (3) highlight the most crucial membrane properties and trends in IEM development; and (4) summarize properties and suitability of different salts for the use in REDHEs, concerning both the regeneration unit and the power unit. The most critical variables in REDHE for power production and solution regeneration are assessed. We first introduce the main performance parameters like resistance and power output. We then discuss the characteristics and requirements of the power unit and the regeneration unit separately, giving an extensive review of the latest literature in the field. Here, special attention is given to tailor-made IEMs for use in RED. In addition, we give an overview of different potential salts for working fluids. The most influential electrolyte qualities concerning the REDHE performance are emphasized, and different salts are compared. We also discuss differences in ionic transport across the IEM among salts.

## 2. Performance Parameters of the RED Stack

When two solutions of different concentrations meet, a liquid junction potential arises. For example, in the case where a selective membrane separates the solutions, as shown in Figure 1, the theoretical electric potential of mixing a concentrate and a diluate salt solution at open circuit conditions is given by [45,46]:

(1) $$\begin{array}{c} \begin{array}{cc} E_{OCV} & =n(E_{m}^{\mathrm{CEM}}+E_{m}^{\mathrm{AEM}})\\ E_{m}^{\mathrm{CEM}} & =\alpha^{\mathrm{CEM}}\frac{RT}{zF}\ln\left(\frac{a_{c}}{a_{d}}\right)=\frac{t_{s}^{\mathrm{CEM}}}{F}\Delta \mu_{s}+\frac{t_{w}^{\mathrm{CEM}}}{F}\Delta \mu_{w}\\ E_{m}^{\mathrm{AEM}} & =\alpha^{\mathrm{AEM}}\frac{RT}{zF}\ln\left(\frac{a_{c}}{a_{d}}\right)=\frac{t_{s}^{\mathrm{AEM}}}{F}\Delta \mu_{s}+\frac{t_{w}^{\mathrm{AEM}}}{F}\Delta \mu_{w} \end{array} \end{array}$$

where the subscript *OCV* stands for *open-circuit voltage*, *n* stands for the number of unit cells, the subscript *m* stands for *membrane*, α is the apparent permselectivity, *R* is the ideal gas constant, *T* is the temperature in Kelvin, *F* is the Faraday constant, *z* is the valence number, and $a_{c}$ and $a_{d}$ are the activities of the concentrated and dilute solution, respectively. The activities are a function of the electrolyte concentration, *c*, and a molar activity coefficient, γ (a=γ·c for a single ion). $\Delta \mu_{w}$ and $\Delta \mu_{s}$ are the chemical potential gradients and salt across the membrane of water, respectively [47]. The salt transport number, $t_{s}$, describes the number of moles of electrolyte transported by the electric current, and the water transport number, $t_{w}$, represents the moles of water brought along with salt migration across the membrane.

The permselectivity for one membrane is typically defined as the measured open-circuit voltage divided by the ideal potential across the membrane:

(2) $$\alpha^{\mathrm{IEM}}=\frac{E_{m}^{\mathrm{IEM}}}{\frac{RT}{zF}\ln\left(\frac{a_{c}}{a_{d}}\right)}=t_{s}+t_{w}\frac{\Delta \mu_{w}}{\Delta \mu_{s}}$$

In the case of an electrolyte consisting of only water and one dissolved salt, the signs of the water transport number and the ratio of chemical potential gradients are such that the last term of Equation (2) is a negative contribution to the permselectivity [47]. To calculate the transport numbers in this manner, a linear regression with a minimum of three permselectivity data points is required. The unit cell potential for complete mixing of HC and LC streams across one AEM and one CEM, $E_{cell}$, is given by [15]:

(3) $$E_{cell}=E_{m}^{\mathrm{CEM}}+E_{m}^{\mathrm{AEM}}-r_{\Omega }\cdot i$$

where $r_{\Omega }$ is the Ohmic resistance of a unit cell (one dilute compartment, one AEM, one concentrate compartment, one CEM) (Ω$m^{2}$) and *i* is the current density [48]. Since typically spacers are used between the membranes to induce better mixing and control the flow, their effect on the Ohmic resistance has to be considered [22]:

(4) $$r_{\Omega }=\frac{r_{AEM}}{\left(1-\beta \right)}+\frac{r_{CEM}}{\left(1-\beta \right)}+\frac{d_{s}}{\rho_{d}\epsilon^{2}}+\frac{d_{s}}{\rho_{c}\epsilon^{2}}$$

where $r_{CEM}$ and $r_{AEM}$ (Ω$m^{2}$) are the Ohmic resistance of the CEM and AEM, respectively, β (dimensionless) is the spacer shadow (the part of the membrane covered by a spacer), $d_{s}$ (m) is the thickness of the spacer, ϵ (dimensionless) is the porosity (i.e., the factor to correct for the occupied volume by the spacer; unity when no spacer is used, <1 with spacer), and $\rho_{c}$ and $\rho_{d}$ (Ωm) is the resistivity of the dilute and concentrate solution, respectively. The power generated by a RED stack, P ($W\cdot m^{2}$), is found by multiplying the cell potential with the current density [15]:

(5) $$P=E_{OCV}\cdot i-nr_{\Omega }\cdot i^{2}$$

Taking the derivative of Equation (5) for the current leads to an expression for the current that we reinsert into Equation (5) to find the peak power density, $P_{d}$ [15]:

(6) $$P_{d}=\frac{E_{OCV}^{2}}{4nr_{\Omega }}$$

In RED applications aiming at withdrawing electricity, rinse solutions containing redox couples are circulated at the electrode surfaces to enable the conversion of chemical potential to electric power. Iron based redox couples (i.e., FeCl^3^/FeCl_2_ and hexacyanoferrate(III)/hexacyanoferrate(II)) are most commonly used (compare Table 2) due to their low toxicity and high stability [16]. The respective redox reactions at the anode and cathode are [15]:

(7) $$\begin{array}{ccccc} \mathrm{Anode}: & {\mathrm{Fe}}^{2+} & ⟶ & {\mathrm{Fe}}^{3+}+e^{-} & E_{0}=-0.77\;V \end{array}$$

(8) $$\begin{array}{ccccc} \mathrm{Cathode}: & {\mathrm{Fe}}^{3+}+e^{-} & ⟶ & {\mathrm{Fe}}^{2+} & E_{0}=0.77\;V \end{array}$$

However, when the aim is to harvest hydrogen from the RED stack, water splitting is induced at the electrodes. For the generation of O_2_ and H_2_ at the electrodes, the pH in the rinse solutions needs to be acidic or alkaline. In alkaline conditions, the respective redox reactions are [49]:

(9) $$\begin{array}{cc} \mathrm{Anode}:\;\begin{array}{c} {\mathrm{OH}}^{-} \end{array} & ⟶\frac{1}{2}\begin{array}{c} H_{2}O \end{array}+\frac{1}{4}\begin{array}{c} O_{2} \end{array}+e^{-}\;E_{0}=-0.401\;V \end{array}$$

(10) $$\begin{array}{cc} \mathrm{Cathode}:\;\begin{array}{c} H_{2}O \end{array}+e^{-} & ⟶\frac{1}{2}\begin{array}{c} H_{2} \end{array}+\begin{array}{c} {\mathrm{OH}}^{-} \end{array}\;E_{0}=-0.828\;V \end{array}$$

(11) $$\begin{array}{cc} \mathrm{Total}:\;\frac{1}{2}\begin{array}{c} H_{2}O \end{array} & ⟶\frac{1}{2}\begin{array}{c} H_{2} \end{array}+\frac{1}{4}\begin{array}{c} O_{2} \end{array}\;E_{0}=-1.229\;V \end{array}$$

## 3. The Solution Regeneration Unit

**Figure 3:**
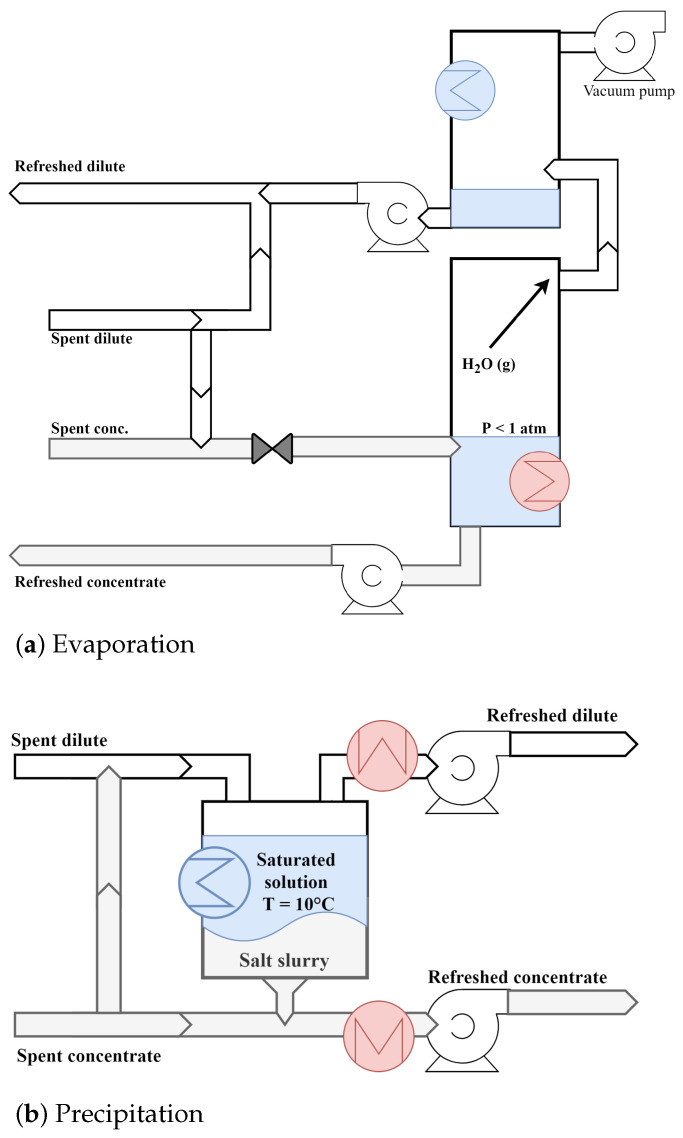
Schematic description of a thermal solution regeneration by (**a**) evaporation and (**b**) precipitation. A red heat exchanger depicts heat added to the system, whereas a blue heat exchanger marks heat withdrawn.

To store energy using salinity gradients, a power unit such as RED is combined with desalination technology for solution regeneration to form a closed loop. External power can be used to increase the concentration difference of the LC and HC stream, charging the system. Energy is then stored in the form of a chemical potential difference [15]. Alternatively, the system can be charged with waste heat, and hydrogen can be produced to store the energy [50]. For the regeneration of the spent salt solutions, two general approaches are possible: (a) solvent-extraction (e.g., distillation and evaporation), as schematized in Figure 3a,b salt-extraction (e.g., salt precipitation), as schematized in Figure 3b [51]. For (a), the solvent is recovered from the outlet HC solution. The extracted solvent is then combined with the outlet LC solution to recover the feed state of both solutions. For (b), the salt is recovered from the outlet LC solution. The extracted salt is then added to the outlet HC solution to recover the feed state of both solutions [52]. The energy efficiency of a REDHE can be described as [30]:

(12) $$\eta =\frac{P}{{\dot{Q}}_{wh}}$$

where ${\dot{Q}}_{wh}$ is the waste heat supplied to drive the regeneration step. Another useful metric for the REDHE performance is the exergy efficiency. Exergy is a measure for the maximum theoretical amount of work obtainable through the conversion of a heat flux into power in a thermodynamic cycle. One major limitation for the closed-loop SGE heat engine is the vast amount of thermal energy needed for the solution regeneration, which is the primary source of exergy destruction [51,53].

Different methods for restoring the initial concentrations using waste heat have been proposed in the literature. Table 1 gives an overview of experimental and theoretical studies on REDHE using different regeneration technologies. Studies categorized as experimental work report values obtained through experimental investigations on a REDHE or parts of it, while studies categorized as theoretical work report values obtained by mathematical models of a REDHE system or parts of it. As can be seen from Table 1, membrane distillation (MD) and multi-effect distillation (MED) are the most popular choices for the regeneration step. Both technologies use evaporative separation processes to extract the solvent from the outlet streams. Alternatives for solvent extraction regeneration technologies are liquid-liquid extraction, azeotropic mixture separation, adsorption/desorption, and absorption/desorption cycles, and extraction by forward osmosis (FO) using temperature-sensitive drawing agents [30]. The salt extraction strategies are limited to two main alternatives: the use of thermolytic salts (e.g., ammonium bicarbonate) [54,55,56,57] and salt precipitation [58,59].

It is apparent from Table 1 that NH_4_HCO_3_ is the most popular salt for use in REDHE. NH_4_HCO_3_ is a thermolytic salt, meaning that it readily decomposes into ammonia (NH_3_) and carbon dioxide (CO_2_) in aqueous solutions upon moderate heating [60,61,62]. Luo et al. proposed a thermally-driven electrochemical generator in 2012 consisting of a RED stack and a thermal separation unit. In this approach, ammonium bicarbonate (NH_4_HCO_3_) was used in a REDHE for the first time. A distillation column powered by waste heat was used to remove NH_4_HCO_3_ from the LC outlet by converting it to NH_3_ and CO_2_, which were recycled into the HC outlet [57]. A power output of 0.33 ${W\;m}^{-2}$ was achieved with an initial concentration difference of 1.3 M. This is the lowest power output reported in Table 1, and it coincides with the thickest spacers used (0.5 mm). Since the spacer thickness directly contributes to the cell resistance (see Equation (4)), efforts are directed towards decreasing the required spacer thickness. In addition, recent studies by Vassallo et al. suggest that air stripping, as used by Luo et al., is not a feasible option for thermolytic salt generation. This is due to the dilution of ammonia and carbon dioxide by the air stripping stream and the concurrent reduction of their partial pressure hindering the absorption step [43]. The comparison of regeneration of thermolytic salt solutions by air and vapor stripping showed specific thermal consumption ranging from 150 kWh/$m^{3}$ to 215 kWh/$m^{3}$ using air stripping, and from 166 kWh/m${}^{3}$ to 290 kWh/m${}^{3}$ using vapor stripping, respectively (for inlet concentrations of the thermolytic salt between 0.25 M and 2 M). The predicted maximum overall exergy efficiency was slightly below 5%. The specific thermal consumption was largely dependent on the inlet concentration of the thermolytic salt and could be improved by using multi-step and optimized regeneration units [43]. Considerably higher exergy efficiency of 24% was predicted by a comprehensive exergy analysis of a REDHE performed by Ortega-Delgado et al., using multi-effect distillation (MED) for solution regeneration. The MED unit was the primary source of exergy destruction [53]. The same is true when using membrane distillation (MD) instead of MED. Since the desired concentration difference between the LC and HC streams is high, MD units typically have high thermal consumption [51]. However, studies by Hu et al. [63] suggest that the energy conversion efficiency obtainable with the MED-RED hybrid power system (1.01%) is lower than that of MD-RED approaches. They further propose to enhance the capturing SGE ability of the RED stack by implementing multiple RED stacks that can either be controlled independently or serially [64].

Tamburini et al. [30] compared the performance of a REDHE with MED and stripping of a thermolytic salt for concentration regeneration, achieving a maximum power output of 7.5 ${W\;m}^{-2}$ with MED and 7.7 ${W\;m}^{-2}$ with stripping. In the respective study, a broad range of different salts are considered as working fluids for the first time, and a comparatively high power yield is predicted.

Salt precipitation is a promising alternative that requires less energy input than solvent evaporation; however, the maximum achievable salinity gradient is limited by the solubility limits of the applied salt. Krakhella et al. modeled hydrogen production and energy requirements for a REDHE at 40 ${}^{\circ }$C with KNO_3_ solution as working fluid, where they compared an evaporation and a precipitation regeneration unit. They reported that at an upper temperature of 40 ${}^{\circ }$C RED with concentrations relevant for the evaporation process, the unit could deliver seven times higher unit cell power density per cross-section area than RED with concentrations relevant for precipitation. Evaporation for regeneration of the spent salt solutions performed better concerning the process cost. The hydrogen production per membrane area is higher; however, the energy demand is significantly lower when using salt precipitation for solution regeneration. The energy consumption allocated to hydrogen production with a precipitation regeneration unit using low-grade waste heat is similar to conventional technologies like proton-exchange membrane water electrolysis and alkaline water electrolysis [52].

**Table 1:** Overview of studies on RED Heat Engines.

Working Fluid $c_{\mathrm{HC}}$/$c_{\mathrm{LC}}$ ($\mathrm{mol}\;L^{-1}$)	Regeneration Unit	RED Stack	$P_{d}$ (W$\;m^{-2}$)	$E_{\mathrm{OCV}}$ (V)	H2 ($g\;h^{-1}\;m^{-2}$)	Ref.
Experimental Work						
NH_4_HCO_3_	air stripping + adsorption		2.42		no	[55,65]
NH_4_HCO_3_	air vs. vapour stripping + absorption/ condensation				no	[43]
NH_4_HCO_3_ 1.5/0.2	distillation column	20 cell pairs Selemion CMV/AMV 10.5 × 7.5 cm${}_{membrane}^{2}$ 130 μm $d_{s}$ 500 μm $d_{m}$	0.33	3.07	no	[57]
NH_4_HCO_3_						
1.5/0		20 cell pairs			yes	[37]
Theoretical work						
NaCl 2/0.01	MD $L_{md}$ = 5 m					[51]
NH_4_HCO 2.4–2.6/ 0.01–0.075	stripping + adsorption		4.8–8.6		no	[65]
NH_4_HCO 2.0/0.5	vapour stripping + adsorption/ condensation				no	[43]
NaCl 3/0.05	MED	1000 cell pairs Fujifilm Type 10 25 × 100 cm${}_{membrane}^{2}$ 150 μm $d_{s}$ 125 μm $d_{m}$	1.9–4.3		no	[53]
NaCl 1–5	MD				no	[66]
NaCl 5/0.05	MED	930 cell pairs Fujifilm 10 × 10 cm${}_{membrane}^{2}$ 120 μm $d_{s}$			no	[63]
NaCl 2–5/ 0.01–0.2	MED	50 cell pairs 10 × 10 (10 × 88) cm${}_{membrane}^{2}$	5.4 (2.9)		no	[67]
KNO_3_	(1) salt precipitation (2) water evaporation	(1) 43–93 cell pairs (2) 15–18 cell pairs Fumatech FAS-50/FKS-50 13 × 9 cm${}_{membrane}^{2}$ 155 μm $d_{s}$ 50 μm $d_{m}$	(1) 0.2–1.0 (2) 3.2–6.5	1.33	(1) 1.1 (2) 2.6	[52]
NH_4_HCO_3_ 0.05–2/0.01	distillation column	5 cell pairs 300 μm $d_{s}$ 120 μm $d_{m}$	0.84		no	[68]
various sol.limit/0.05	(1) MED (2) thermolytic salt (NH_4_HCO_3_)	10 cell pairs Fujifilm 10 × 10 cm${}_{membrane}^{2}$ 270 μm $d_{s}$ 125 μm $d_{m}$	(1) 7.5 (2) 7.7		no	[30]

c_HC_—concentration of the HC stream, c_LC_—concentration of the LC stream, *P_d_*—peak power density, *E_OCV_*—open circuit potential, MD—membrane distillation, MED—multi-effect distillation, *L_md_*—membrane distillation channel length, *d_s_*—spacer thickness, *d_m_*—membrane thickness.

## 4. Membranes and RED Stack Design

Ion-exchange membranes are the key components in all electro-membrane processes as their properties determine to a large extent the system performance. In general, IEMs are obtained by introducing charged moieties onto polymer backbones. For example, anion-exchange membranes contain positive fixed charges that make them preferentially permeable to anions. At the same time, cations are excluded to a large extent and vice versa for cation-exchange membranes [69].

The properties of IEM material involving water uptake, ion-exchange capacity (IEC), and fixed charge density (FCD) are of great importance to determine the performance of RED in terms of permselectivity and electrical resistance. For instance, water uptake plays a significant role in controlling the dimensional stability of the membrane and its resistance [70]. Thus, even though high water content offers low membrane electrical resistance providing high energy-efficient operation, it tends to lower the permselectivity. The water uptake of an IEM can be quantified by [71]:

(13) $$\mathrm{Water}\;\mathrm{uptake}=\frac{W_{\mathrm{wet}}-W_{\mathrm{dry}}}{W_{\mathrm{dry}}}\cdot 100$$

where $W_{\mathrm{wet}}$ and $W_{\mathrm{dry}}$ are the weight of IEM in the wet and dry phase, respectively.

IEC is a measure of the number of fixed charged groups within the membrane matrix. It is determined in milli-equivalents (meq) of charged groups per gram of dry membrane [69]. For charging CEMs, sulfonic (−SO_3_^2−^) and carboxylic (−COO^−^) acid groups are most commonly introduced in the membrane matrix, while ammonium (−NH_4_^+^) groups are most frequently used to charge AEMs. A higher IEC of the membrane network can provide a higher permselectivity of the membrane. However, higher charge density induces membrane swelling due to the hydration capability of the charged units [72]. Therefore, it is essential to balance the number of charged units and the hydrophobic domain of the membrane network for improved performance.

The FCD is defined as the milli-equivalents of charged groups per gram of water in the membrane (meq/g H_2_O), and can be calculated by the ratio of IEC and water uptake of the membrane [73]:

(14) $$\mathrm{FCD}=\frac{\mathrm{IEC}}{\mathrm{Water}\;\mathrm{uptake}}\cdot \rho_{w}$$

where $\rho_{w}$ is the density of water. The FCD is a vital parameter for optimizing membrane permselectivity and resistance by adjusting IEC and water uptake, which controls the membrane’s charge density and swelling properties. In addition, the membrane properties are sensitive to the type and concentration of salt. Therefore, it is essential to adjust the electrolyte-membrane system to achieve the maximum power output for a REDHE.

RED is typically considered for seawater and river water as feed solutions due to their availability in large volumes. Consequently, many studies have been performed with NaCl solutions that mimic the concentrations of these naturally occurring streams. The power density, an important performance parameter for the RED system, can be enhanced by optimizing operating parameters like spacer thickness and geometry, hydrodynamic conditions, and salinity gradient and developing more specialized membranes [74]. Table 2 gives an overview of studies published on RED for power generation using commercial membranes, showing the applied operating conditions and the yielded power density, stack resistance, and OCV.

The increased interest in RED technology has been followed by rising efforts to synthesize custom-made membranes tailored to maximize power generation. In electrochemical membrane applications, membrane characteristics mainly depend on the amount of charged species groups and their distribution within the membrane matrix. Table 3 gives an overview of the different methods that have been proposed to provide tailor-made AEMs and CEMs for RED applications with the desired cationic and anionic moieties, respectively.

The presence of multivalent ions like Mg^2+^ in natural waters reduces the power output of RED as they are transported against their concentration gradient, a phenomenon know as *uphill transport* [82,83,84,85]. Therefore, efforts have been made to prepare membranes with high selectivity for monovalent ions, both concerning AEMs [86,87] and CEMs [76,88,89].

Apart from featuring low resistivity and high permselectivity, feasible membranes must be easy to prepare and cost-competitive. Therefore, the polymers used as the membrane backbone must be functionalized easily to bind the main chains with charged groups. Other aspects considered in membrane preparation are the polymer preparation processes, which should be easily controllable and not release toxic substances. Furthermore, the polymeric materials themselves should be affordable, especially for industrial-scale applications. Three commonly used low-cost IEM materials are polyvinyl alcohol (PVA), poly(2,6-dimethyl-1,4-phenylene oxide) (PPO), and polyvinylchloride (PVC) [90,90].

Power density is directly proportional to the square of permselectivity while inversely proportional to the area resistance (see Equations (2) and (5)), indicating that IEMs with higher permselectivity and lower area resistance tend to achieve higher power density [91]. The preparation conditions are significant in the determination of membrane properties. Providing high IEC is one way of improving the permselectivity of the IEMs. However, too many fixed charged groups cause swelling of the membranes, which lowers the number of functional groups used by ions. This, in turn, results in lower permselectivity due to inefficient donnan exclusion. Membranes can be reinforced with specific materials against swelling, but in this case, they tend to be thicker, which increases electrical resistance, thereby decreasing power density [91,92]. As a result of counteracting effects of the membrane properties on power density, network and surface properties of the membranes are of great significance to ensure high performance. The inherent properties of the polymeric material and the membrane microstructure can be adjusted by suitable membrane preparation methods.

Based on the structure and preparation method, IEMs can be classified as homogeneous and heterogeneous. Homogeneous membranes display an even distribution of the charged groups, whereas heterogeneous membranes feature a clustered and uneven distribution of the mixed ionic moieties within the membrane network [69]. There are several techniques to prepare such IEMs to be used in RED systems. For example, solution casting followed by solvent evaporation is one of the techniques to produce dense homogeneous IEMs in which films are formed from functionalized polymers. Güler et al. synthesized both AEMs and CEMs from PECH and SPEEK polymers via solvent evaporation. The power density was improved from 0.9 to 1.28 $W\;m^{-2}$ with the decrease of membrane area resistance from 2.05 to 0.82 Ω${\mathrm{cm}}^{2}$ by decreasing the membrane thickness. However, no correlation was found between permselectivity and power density [93,94]. RED membranes can be manufactured by grafting polymers with functional ionic moieties to adjust the polymeric material’s charge density and swelling behavior. Cho et al. applied solvent evaporation to prepare homogeneous IEMs using different materials with different swelling behaviour. The membrane with the lowest swelling degree resulted in the highest permselectivity, thereby yielding the highest power density (1.2 $W/m^{2}$) [95]. Solvent evaporation can also be used to prepare composite membranes by blending or embedding charged inorganic particles into the polymer network, followed by membrane casting [96,97,98]. For example, Hong et al. embedded SiO_2_-SO_3_H in a variety of sizes to adjust membrane properties. Membranes with larger fillers displayed relatively higher IEC and lower area resistance ($0.85\;\Omega {\mathrm{cm}}^{2}$), thereby showing the highest gross power density of 1.3 $W\;m^{-2}$ [96].

IEMs can further be synthesized by pore-filling techniques in which monomeric/polymeric electrolytes are impregnated onto a porous substrate [99,100,101]. Pore-filling membranes PCEM and PAEM represented much lower area resistance (0.42 Ω${\mathrm{cm}}^{2}$) than Fujifilm type commercial CEMs (2.10 Ω${\mathrm{cm}}^{2}$) and AEMs (1.22 Ω${\mathrm{cm}}^{2}$), thereby displaying a higher power density of 1.95 $W\;m^{-2}$ compared to commercial membranes (1.46 $W\;m^{-2}$) [99]. Despite the same IEC values of pore-filling and commercial membranes, the swelling degree of pore-filling membranes was lower due to a more hydrophobic character of electrolyte polymers restrained by porous substrates. This provides reasonable mechanical stability without needing additional reinforcement of the membrane, which increases the area resistance.

Apart from tailoring the membrane network, surface modification of existing IEMs is considered one of the most convenient methods for enhanced power density. It is reported that power generation decreases in the presence of multivalent ions [87]. Considering this, Gao et al. developed monovalent-anion selective membranes by layer-by-layer deposition of polyelectrolytes on the membrane to reject divalent sulfate ions. As a result, the gross power density was improved by up to 17% compared to standard AEMs [87]. Tufa et al. developed monovalent CEMs based on polypyrrole-chitosan composites to ease the negative impact of multivalent cations on power density generation. Providing a rigid and tight structure after polymerization on the surface of the CEM restricted the transport of Mg^2^+ and increased monovalent selectivity. The maximum power density with modified membranes of 1.5 $W\;m^{-2}$ presents more than 42% enhancement compared to pristine membranes [88].

Developing suitable membrane preparation techniques specialized for RED applications is of paramount importance to decrease the membrane area resistance to a minimum and consequently optimize the power output. The membrane synthesis determines its morphology, i.e., thickness and tortuosity, making it a critical factor for ionic mass transport through the IEM. Additionally, the inherent polymer hydrophobicity determines the membrane’s resistance towards swelling [102].

**Table 3:** Overview of custom-made IEMs for RED applications (The membrane denominations are taken from the respective studies).

Membrane	Preparation Technique	$d_{m}$ (μm)	Area (cm^2^)	IEC ($\mathrm{meq}\cdot g^{-1}$)	Water Uptake (%)	FCD ($\mathrm{meq}\cdot L^{-1}$ H_2_O)	Area Resistance (Ω cm^2^)	Feed Solution	α	$P_{d}$ ($W\;m^{-2}$)	Ref.
Fuji CEMT1- PPyCS-0.05	Surface polymerization on commercial membrane	122	18	1.7	47.4	3.5	2.12	NaCl 4 M/ 0.5 M	-	1.5	[88]
SPES-P SPES-D	Phase inversion	83 63	207	1.15 1.19	67.2 28.0	1.7 4.3	1.4 1.9	NaCl 4 M/ 0.1 M	<0.5 <0.8	3.64 3.92	[92]
PCEM PAM	Pore filling of porous polyethylene by single impregnation in a roll-to-roll process	16 17	19.6	1.80 1.81	49.5 39.3	-	0.42 0.40	NaCl 0.5 M/ 0.017 M	0.957 0.924	1.95 1.46	[100]
PErC(5)QPS- QPPO	Chemical crosslinking of polyethylene support	51	25	1.2	37	-	0.69	NaCl 0.599 M/ 0.00856 M	-	1.82	[103]
UTFCS- 5/CMX	Spin coating on ceramic support	45	-	-	-	-	1.2	sea/ river water	0.886	0.036	[104]
AEM	Chloromethylation and quaternization of the grafted copolymer films	-	-	1.1 2.9	-	-	0.6	-	-	0.8– 0.9	[105]
PAEM- AA25	Pore filling of polyethylene by photoinduced radical polymerization	17	19.6	1.67	93.72	-	0.323	NaCl 0.5 M/ 0.017 M	0.955	1.50	[99]
PPO-PVA PDDA-PVA	Solution casting and solvent evaporation	50 55	-	1.58–1.91 0.97–1.50	46–93 100–148	1.74–4.2 1.0–1.2	1.30–1.54 0.71–1.32	NaCl 0.5 M/ 0.017 M	0.810–0.873 0.420-0.595	0.25–0.46 0.21–0.46	[106]
PDDA-PVA	Solution casting and solvent evaporation	55	36	1.0–1.54	171–179	-	0.76–1.34	NaCl 0.5 M/ 0.017 M	0.42–0.62	0.34–0.58	[91]
CJMA- 2–7.5	Layer-by-layer deposition of polyelectrolyte	102.7	36	-	-	-	3.1	NaCl 0.51 M/ 0.017 M	0.91	<0.39	[87]
E2C1- DMA0.5	Pore-filled polyethylene by the addition of electrolytes	25	19.6	1.40	-	8	0.754	NaCl 0.5 M/ 0.017 M	0.938	1.524	[107]
PAES-ABCO PAES-IMD PAES-TMA	Solution casting Solvent evaporation Quaternization	64–70 59–64 58–70	34	1.2–1.48 1.19–1.48 1.17–1.45	11–17 8–13 15–30	10.55–12.62 13.31–16.40 6.68–9.06	1.59–3.82 1.65–3.86 1.45–3.53	NaCl 0.5 M/ 0.017 M	0.935–0.972 0.944–0.986 0.916–0.966	1.16 1.2 1.14	[95]
sPVA (2–10%)	Hybrid membrane by solution casting and solvent evaporation	50	36	1.6–2.05	45–75	2.0–4.5	1.3–2.1	NaCl 0.5 M/ 0.017 M	0.80–0.86	0.3–0.462	[108]
SPPO-(0.1–0.8) O-MWCNT	Blending	47–70	36	1.77–2.28	37.6–42.6	4.6–5.5	0.45–0.67	NaCl 0.5 M/ 0.017 M	0.899–0.953	0.37–0.48	[109]
A-SPPO	Ion channel alignment by pulse electric field	80-91	20	0.91-1.06	-	-	0.86	NaCl 0.599 M/ 0.017 M	0.962	1.34	[110]
KIER	Pore filling	26–27	19.6	1.42–2.6	21.7–26.9	6.5–9.8	0.28–0.72	NaCl 0.58 M/ 0.017 M	0.918–0.992	<2.5	[101]
sPPO-SiO_2_– SO_3_H	Solvent evaporation	30	-	0.78–1.18	21–34	2.6–94.7	0.85–1.87	NaCl 0.5 M/ 0.017 M	0.791–0.865	1.3	[96]
Fe_2_O_3_– SO_4_/sPPO	Two-step phase inversion	30–150	36	0.98–1.42	16–58	2.0–6.4	0.82–2.26	NaCl 0.5 M/ 0.017 M	0.771–0.923	0.62–1.4	[97]
Fe_2_O_3_– SO_4_/sPPO	Solution casting Solvent evaporation	100	36	0.87-1.40	20–26	3.4–5.4	0.87–2.26	NaCl 0.5M/ 0.017M	0.686–0.877	1.30	[98]
Flat Ridges Waves Pillars	Solution casting/ Solvent evaporation	190 199 200 212	100	-	-	-	2.55 3.16 2.94 3.20	NaCl 0.507 M/ 0.017 M	0.905 0.896 0.895 0.901	1.10 1.10 1.25 1.30	[111]
SPEEK PECH	Solution casting Solvent evaporation	33–130	100	1.23–1.76	23–54	3.4–5.3	0.82–2.05	NaCl 0.507 M/ 0.017 M	0.891–0.953	1.07–1.28	[93]
PECH	Solution casting/ amination reaction	33–130	100	1.31–1.88	32.2–53.5	3.4–4.1	0.82–2.05	NaCl 0.507 M/ 0.017 M	0.792–0.903	0.90–1.27	[94]

*d_m_*—membrane thickness, *IEC*—ion-exchange capacity, FCD—fixed charge density, *a*—permselectivity, *P_d_*—peak power density.

**Figure 4:**
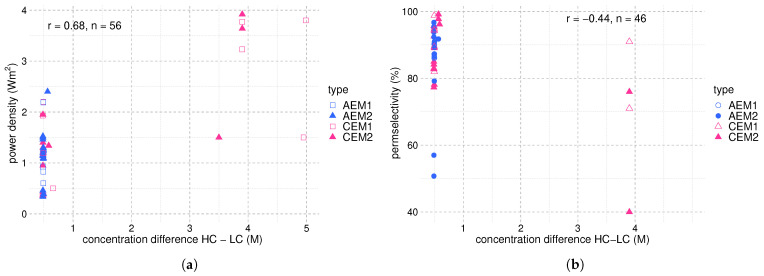
Power density and permselectivity plotted against the concentration gradient across the IEMs in RED studies reported in Table 2 and Table 3 for NaCl. Additional data concerning commercial membranes have been collected from [92,93,112]. A total of 81 observations form the basis of the analysis, with 21 observations of commercial AEMs (AEM1), 20 of tailor-made AEMs (AEM2), 23 of commercial CEMs (CEM1), and 17 of tailor-made CEMs (CEM2). The Pearson coefficient *r* indicates the correlation between the two plotted variables, and *n* refers to the number of observations used for each plot. (**a**) power density vs. concentration difference and (**b**) permselectivity vs. concentration difference.

**Figure 5:**
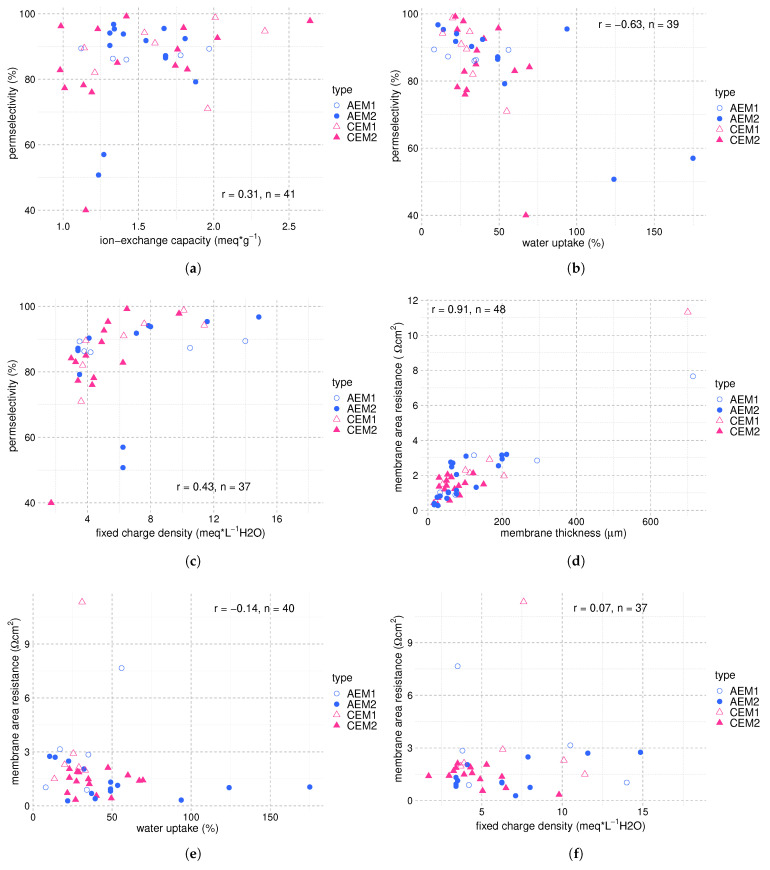
Membrane area resistance and permselectivity plotted against membrane thickness, IEC, water uptake, and FCD. Data are extracted from Table 2 and Table 3, and were restricted to studies working with NaCl. Additional data concerning commercial membranes have been collected from [92,93,112]. A total of 81 observations form the basis of the analysis, with 21 observations of commercial AEMs (AEM1), 20 of tailor-made AEMs (AEM2), 23 of commercial CEMs (CEM1), and 17 of tailor-made CEMs (CEM2). The Pearson coefficient *r* indicates the correlation between the two plotted variables, and *n* refers to the number of observations used for each plot. (**a**) permselectivity vs. ion-exchange capacity, (**b**) permselectivity vs. water uptake, (**c**) permselectivity vs. fixed charge density, (**d**) membrane area resistance vs. membrane thickness, (**e**) membrane area resistance vs. water uptake, and (**f**) membrane area resistance vs. fixed charge density.

**Figure 6:**
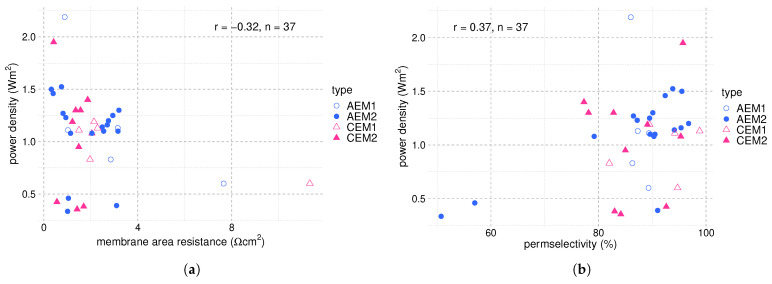
Power density plotted against membrane area resistance and permselectivity for a concentration difference of NaCl of 0.48 to 0.49 M between the HC and LC streams. Data are extracted from Table 2 and Table 3. Additional data concerning commercial membranes have been collected from [92,93,112]. A total of 42 observations form the basis of the analysis, with seven observations of commercial AEMs (AEM1), 18 of tailor-made AEMs (AEM2), 7 of commercial CEMs (CEM1), and 10 of tailor-made CEMs (CEM2). The Pearson coefficient *r* indicates the correlation between the two plotted variables, and *n* refers to the number of observations used for each plot. (**a**) power density vs. membrane area resistance and (**b**) power density vs. permselectivity.

A model considering more efficient membranes than the ones available to date, but with properties that have already been obtained in laboratory studies, predicted a maximum power density of 18 ${W\;m}^{-2}$ for RED using NaCl solutions with ten cell pairs of 10 cm × 10 cm big membranes and MED as regeneration step. The most influential assumptions were permselectivities of 90% for both AEM and CEM, an electrical resistance of 0.5 $\Omega {\mathrm{cm}}^{2}$ per membrane, and a membrane thickness of around 50 μm [30]. Table 3 shows that these characteristics have already been achieved individually for various tailor-made membranes. Yet, the maximum power density reported for lab-scale RED applications has not exceeded 4 ${W\;m}^{-2}$ [92]. The discrepancy between the ideal case and laboratory experiments originates from trade-offs between membrane properties and process conditions. To develop high-performance IEMs, we need to understand the interplay between the prominent membrane and process parameters and their implications for the process performance. We elucidate correlations in the following by interpreting the data gathered in Table 2 and Table 3. Figure 4, Figure 5 and Figure 6 present scatter plots that visualize correlations between different membrane properties and process conditions. In addition to the studies cited in the tables, data concerning commercial membranes has been obtained from studies by Güler et al. [93], Kingsbury et al. [112], and Avci et al. [92]. We calculate the degree of linear correlation between two variables with the Pearson correlation coefficient, *r*, which is the ratio between the co-variance of two variables and the product of their standard deviations [113,114]. When discussing the correlations, it is important to remember that all parameters underlie variations from study to study. Therefore, the scatter plots can only hint at correlations between two variables and are used to structure the discussion. Controlled studies varying one parameter at a time are needed for confirmation. Figure 4a shows a correlation of 0.68 between the concentration difference and the power density. The influence of the solution concentration of the HC and LC feed on the system performance has been one of the most investigated topics in the literature on RED, since it has a direct effect on the power output (see Equations (1) and (5)), but also the cell resistance (see Equation (4)). Therein lies a major trade-off in enhancing the RED performance; decreasing the concentration of the LC stream increases $E_{OCV}$ by increasing the concentration difference between LC and HC streams but concomitantly increases the electrical resistance of the dilute solution. Tedesco et al. confirmed this behaviour for the first RED pilot plant for SGE production; increasing the conductivity of the LC stream not only led to a reduction in stack resistance but also in $E_{OCV}$ due to the lower salinity gradient across the IEMs [21]. Jin et al. developed a two-dimensional multi-physical model for RED that suggests an LC stream contribution of 70% to the total cell pair resistances for HC and LC stream concentrations close to seawater and river water, respectively [115]. However, other studies suggest a much higher contribution from the membranes to the total Ohmic resistance. A model of hydrogen production with REDHE using KNO_3_ indicated that the membrane resistance constituted 98% of the total Ohmic resistance when using concentrations relevant for the precipitation process. However, employing concentrations relevant for the evaporation process reduced the share of the membranes in the Ohmic resistance to 70% [52]. This is logical when considering that for the evaporation process, the resistance of the dilute solution is significantly higher than for the precipitation process; therefore, contributing to a higher fraction of the total Ohmic resistance. Studies by Długołęcki et al. confirm the concentration dependence of the resistance contributions; for a CMX cation exchange membrane, the membrane resistance dominated in 0.5 M NaCl but got surpassed by the solution resistance in 0.017 M NaCl [116]. Ortiz-Imedio et al. developed a model predicting the SGE performance of RED under different operating conditions that highlight the dominance of the LC compartment resistance and suggest that working at the highest possible salinity gradient doesn’t necessarily provide the highest possible process performance [77]. Efforts to decrease the Ohmic resistance of the RED stack have been made by altering the process design (i.e., using profiled membranes or multi-step RED). Kim et al. compared the power generation of two RED stacks with different dimensions (5 × 5 cm${}^{2}$ and 15 × 15 cm${}^{2}$), and found that the non-Ohmic contribution to the internal resistance increased significantly with stack size. They further suggest that the composition of pore-filling IEMs has a significant affect on the non-Ohmic resistance of a RED stack [117]. Furthermore, the presence of non-conductive spacers significantly increases the resistance and lowers the power output in RED by blocking parts of the membrane [118,119,120,121], as reflected by Equation (4). Güler et al. [111] prepared micro-structured membranes that create channels for the feed water and render spacers obsolete. Straight-ride, wave and pillar structured membranes were used facing the LC compartments of the RED stack where the electrical resistance is the highest. The pillar structured membranes showed the best performance with 38% higher gross power density and 20% higher net power density as compared to the setup featuring flat membranes with non-conductive spacers [111]. Different profile geometries can optimize the hydrodynamic flow and obtain more efficient mixing than conventional spacers. Experimental data from Vermaas et al. [122] show that RED stacks with profiled membranes give much lower hydraulic friction than stacks with traditional non-conductive spacers, allowing higher Reynold numbers. Therefore, micro-structured membranes can improve the gross power output, especially when high Reynold numbers are employed. In another study, Vermaas et al. investigated the effect of the intermembrane distance on the power density achieved with RED, varying the spacer thickness from 60 to 485 μm. They showed that the Ohmic resistance was dominated by the resistance of the LC compartment, which increased proportionally with the intermembrane distance. Small intermembrane distances decrease the Ohmic resistance and thereby the total internal resistance substantially. Therefore, the gross power density increases with decreasing intermembrane distance. The maximum power density of 2.2 $W\;m^{-2}$ was achieved with the minimum intermembrane distance. The energy efficiency was also positively influenced by minimizing the intermembrane thickness. The authors suggest that efforts in establishing RED energy systems should be directed towards developing spacer-less designs to reduce the pressure drop required to pump the feed-water through the module [81]. Despite of this, small intermembrane distances cause considerable hydraulic friction and can require pre-treatment of the solution to avoid fouling. Furthermore, the significant pressure drop for small intermembrane spaces sets a boundary for the maximum applicable flow rate. Tsai et al. have shown that the power output also increases with reduced channel length as a result of reduced resistance [123].

Figure 4b shows a negative correlation between concentration difference and permselectivity. A detrimental effect of increased concentration gradients on permselectivity has been observed in various studies [80,85,124]. Daniildis et al. [80] performed RED stack experiments applying a wide range of salinity gradients, with the LC ranging from 0.01 to 4 M NaCl and the HC ranging from 0.5 to 5 M NaCl. In accordance with Equation (1), they observed an increase in the power density with the salinity gradient. Despite this, the energy efficiency was higher for feed streams with low salt concentrations and low salinity gradients. This was ascribed to cumulative ionic shortcut currents, losses due to water transport, and, to a smaller extent, salt transport through the membrane. The highest power density was found for the most extreme salinity gradient (0.01/5 M NaCl), despite the lowest permselectivity for this combination.

Figure 5 summarizes the influence of the membrane properties IEC, water content, and FCD on the permselectivity and membrane resistance. The IEC shows a weak positive correlation while the water uptake shows a medium negative correlation with the permselectivity (see Figure 5a,b). Higher IEC allows for higher ionic concentrations within the membrane, and thus, a higher cell voltage. The decrease in permselectivity with increased water uptake is in good agreement with Equation (2); a higher swelling degree facilitates water transport, which has a detrimental effect on the permselectivity. The fixed charged density is a function of both IEC and water uptake (see Equation (14)) and shows a correlation of 0.43 with the permselectivity among the reviewed studies (Figure 5c). A higher FCD causes an increase of the total ionic concentration in the membrane matrix, generating a growth of ionic current which increases the permselectivity.

Membrane thickness, fixed charge density, and water uptake are also associated with the membrane’s ionic resistance [70,92,125]. Figure 5d suggests a strong correlation between the membrane thickness and the membrane area resistance, with an r-value of 0.91 for 48 observations. Interestingly, Figure 5e,f show no correlation of FCD and water uptake with membrane area resistance, with r-values of −0.14 and 0.07, respectively. This can partially be ascribed to the variation around one order of magnitude in membrane thickness among the studies being the dominant factor for differences in membrane resistance. Additionally, the inherent properties of the polymeric material, namely its hydrophobicity, determine the area resistance to a large extent. Therefore, the low correlation of water uptake and FCD with membrane area resistance among the studies can hint at the importance of the polymeric material for membrane performance. In accordance, findings by Kingsbury et al. suggest that the salt and water permeation through IEMs is mainly governed by the membrane microstructure rather than chemical interactions with the polymer chain in highly swollen IEMs [112]. Consequently, for the development of high-performance IEMs, it is crucial to elucidate the physicochemical properties of the membrane polymers that determine the variation in ion transport numbers and membrane area resistance.

Since the concentration difference is such a dominant parameter for the power density, we illustrate the power density as a function of the membrane area resistance and the permselectivity at similar concentration differences to get a more meaningful analysis. The respective plots are shown in Figure 6. As expected, a negative correlation exists between the power density and the membrane area resistance and a positive correlation between the power density and the permselectivity. The correlation factors are weak, which underlines the complex interplay between the different variables.

Apart from the concentration difference between LC and HC streams, other operating conditions investigated are the flow velocity of the inlet streams and the solution temperature. The flow velocity of the LC stream was found to have a marginally positive impact on the potential at low speed [21,126], but the power output and conversion efficiency was shown to increase by 2.3 times and 10%, respectively, at high rate when convection effects become significant compared to diffusion [127]. Krakhella et al. compared the performance of a RED stack at two different temperatures (i.e., 25 ${}^{\circ }$C and 40 ${}^{\circ }$C). Within this temperature range, no significant impact of temperature on $E_{OCV}$ and power density was observed. The authors proposed raising the temperature above their applied upper limit of 40 ${}^{\circ }$C to investigate the temperature dependency further. They expect a positive impact of higher temperature on the membrane permselectivity and resistance [15]. Indeed, a positive effect of temperature on membrane resistance has been shown experimentally through electrochemical impedance spectroscopy [128]. Raka et al. report decreasing membrane resistance when increasing the operating temperature of a RED stack from 22 ${}^{\circ }$C to 40 ${}^{\circ }$C, which is ascribed to increased ion mobility [129].

Since NaCl is the by far most popular salt to use in RED (compare Table 2 and Table 3), most commercial and custom-made membranes are designed for NaCl. It should be noted that the physical properties of the membrane are strongly dependent on the salt used; therefore, ion-selective membranes should be designed to target specific ions to exploit SGE with RED using non-conventional salts. Zhu et al. tested the power density obtainable with a RED stack firstly with different NaCl concentrations, and secondly with NH_4_HCO_3_ instead of NaCl, using commercial membranes. They found that the performance of the RED stack was lower using NH_4_HCO_3_ for the same molar concentrations of both salts. This is ascribed to lower ion activities for NH_4_HCO_3_; however, when the solution conductivities were matched, both salts performed similarly [54]. Membranes designed for use with NH_4_HCO_3_ solutions could enhance the mass transport of this salt. Krakhella et al. compared the ion conductivities of KNO_3_ and NaCl in commercial AEMs and CEMs. The ion conductivity of KNO_3_ was similar to that of NaCl in the CEMs but significantly lower in the AEMs [52]. Since the REDHE is operated as a closed-loop system, salts different from NaCl could be more feasible working fluids. To tailor-make IEMs for optimized hydrogen production and heat requirements for REDHE, the most promising electrolytes must be identified based on their ionic transport properties in the RED stack and their regeneration ability.

## 5. Potential Salts for REDHE

As in all electrochemical processes, the performance of RED varies strongly with the properties of the electrolyte solution. The salt solution recycled in the closed-loop system is of paramount importance for energy generation and solution regeneration efficiency. Fundamental salt properties to consider for use in REDHE include

- The solubility in water defines the maximum concentration difference achievable; therefore, the maximum driving force for energy generation. A salt with high solubility and a high temperature dependency of the solubility is favorable for use in a REDHE. For precipitation as the solution regeneration step, a high temperature dependency of the solubility is crucial for maximizing the power output [30,52]. NaCl has a moderate solubility at room temperature, and the temperature dependency of the solubility is low. This is sub-optimal for use in a REDHE.
- The equivalent conductivity of the aqueous solution determines to a large extent the stack resistance and should be high to minimize the resistance [79]. NaCl has a relatively high conductivity in an aqueous solution compared to other salts [30].
- The activity coefficient ratio has a strong influence on the generated open cell potential within the RED unit [30], as reflected by Equation (1).
- The dissolution enthalpy change of a salt plays a significant role in the heat requirement of solution regeneration via precipitation, and thus for the process efficiency. Salts can have a positive or negative enthalpy change of dissolution, increasing or decreasing the heat requirement, respectively [130,131].
- The affinity and mobility of ions in the IEM determine their permselectivity; therefore, it influences the achievable power output. Affinity and mobility are functions of ion properties like hydrated radius and hydration energy.

**Figure 7:**
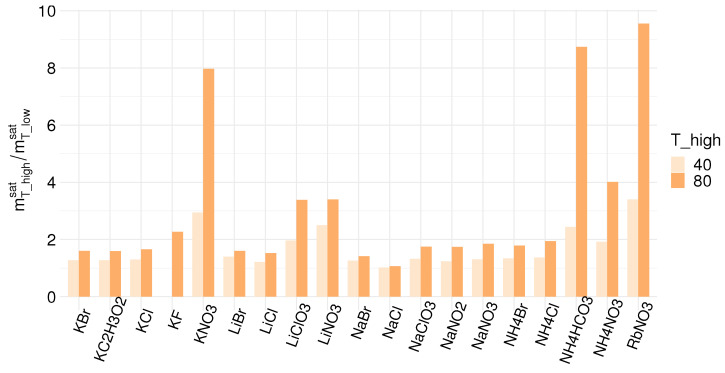
Saturation concentration ratio for aqueous solutions of the salts listed in Table 4 between 40 ${}^{\circ }$C and 10 ${}^{\circ }$C (light orange), and 80 ${}^{\circ }$C and 10 ${}^{\circ }$C (dark orange). Note that there was no data available for the saturation concentration of KF at 40 ${}^{\circ }$C.

The concentration of the HC solution is a function of the salt solubility at a specific operating temperature. For precipitation of salt, the concentration of the dilute solution is limited by the salt solubility at the cooling temperature, while for evaporation as a regeneration step, there is technically no limit to the lower concentration. Critical properties of different eligible salts for SGE are summarized in Table 4. The salt solubility in an aqueous solution is given at three temperature levels; 10 ${}^{\circ }$C, 40 ${}^{\circ }$C, and 80 ${}^{\circ }$C. The lowest temperature level is chosen as the base temperature the solutions can be cooled down to. This depends on available cooling streams, e.g., seawater, and varies with the geographical location of the technology. Most commercial membranes can withstand temperatures up to 40 ${}^{\circ }$C. In contrast, specialized membranes operate at up to 80 ${}^{\circ }$C. Therefore, we chose a temperature of 10 ${}^{\circ }$C for the recovered solution, while for the spent solution, we compare the system performance at 40 ${}^{\circ }$C and 80 ${}^{\circ }$C. Figure 7 shows the change in saturation concentration for the respective salts between a lower temperature of 10 ${}^{\circ }$C and upper temperatures of 40 ${}^{\circ }$C and 80 ${}^{\circ }$C. The concentration ratio of the HC and LC stream has immediate implications for the power output of the RED stack, as suggested by Equation (1). The higher the concentration difference, the higher the open circuit potential.

The concentration difference between the HC and the LC stream available when using precipitation is determined by the temperature dependency of the solubility. A significant change in solubility between the lower and upper temperatures used in a given application corresponds to a high driving force for hydrogen production. Figure 7 shows that NH_4_HCO_3_, NH_4_NO_3_, RbNO_3_ and KNO_3_ exhibit the most extensive changes in solubility when the temperature is increased, especially to 80 ${}^{\circ }$C; however, the concentration difference between the LC and HC streams is not the only parameter that determines the hydrogen output and it is vital to consider all relevant factors. As discussed in Section 4, the comparatively low ionic conductivity of NH_4_HCO_3_ reduces its feasibility for use in RED power production [54]. Low conductivities and a low activity coefficient ratio can offset the benefits of high concentration differences at the two temperature levels, resulting in low hydrogen production. It is worth noting that all of these factors are functions of the temperature (i.e., the temperature range employed). Unfortunately, conductivity data for salts at different temperatures are not readily available and require further experimental studies.

**Figure 8:**
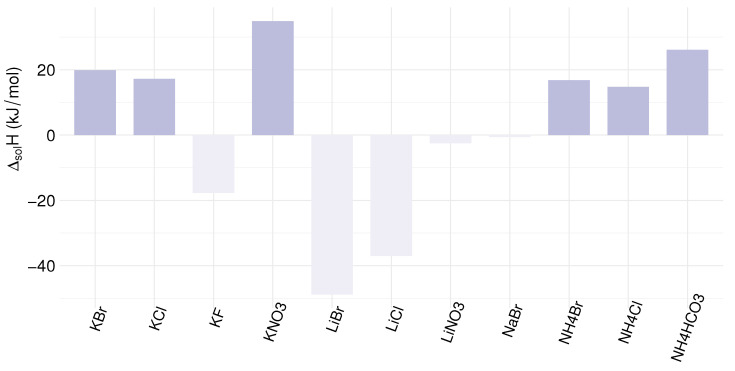
Enthalpy of dissolution for various salts at infinite dilution. The dark purple bars represent salts with an endothermic enthalpy of dissolution, while the light purple bars represent salts with an exothermic enthalpy of dissolution. Data extracted from [132].

The advantage of the precipitation process is the lower energy requirement to regenerate the electrolytes. For precipitation, most of the energy needed is for heating the solution and dissolving the salt. There is a big difference in heat requirement for endothermic versus exothermic salts. Figure 8 shows the enthalpy of dissolution for the selected salts. Salts with endothermic dissolution enthalpy have a higher heat requirement for regeneration, as heat is needed for dissolution. On the other hand, salts with exothermic dissolution enthalpy only require heat input for increasing the electrolyte temperature from 10 to 40 ${}^{\circ }$C after renewing the solution concentrations. The molar enthalpies of solutions at infinite dilution for the considered salts are listed in Table 4. The three salts KF, LiNO3 and LiBr have especially endothermic solution enthalpies and may prove to be excellent electrolytes for REDHE. For evaporation, the evaporation of water from the concentrated solution is the major source of energy consumption. The membrane resistance constitutes a large part of the overall unit cell resistance in both cases. Both the dilute and concentrate solutions are kept at relatively high concentrations when utilizing the precipitation method. As a consequence, the electrolyte resistances are generally low for almost all of the investigated salts. LiCl is an exception here due to the anomaly that the salt displays low conductivities at high concentrations [133]. For the evaporation method, the dilute is commonly kept at below 1 mol kg${}^{-1}$, giving a significant resistance contribution.

Wu et al. [134] studied the electrical conductivity of LiCl, LiBr and LiI to assess their feasibility for use in a REDHE with a MED regeneration unit. A methanol-water mixture was proposed as solvent due to favourable electrochemcial and thermodynamic properties. LiI solutions showed the highest conductivities among the three tested salts, and using methanol as a solvent yielded higher conductivies than water. Giacalone et al. [44] analysed a set of salt solutions that are not typically used in RED with regards to their thermodynamic eligibility. Their research suggests that potassium acetate (KAc), caesium acetate (CsAc) and LiCl are promising candidates for closed-loop RED systems, mainly due to their remarkably high solubility in aqueous solution compared to NaCl, paired with high conductivities. Tamburini et al. modeled the performance of a RED unit using different salts and concluded that a variety of salt solutions have the potential to obtain a significantly higher maximum power density than NaCl. The highest maximum power density of almost 40 $W\;m^{-2}$ was achieved with a LiBr solution [30].

Based on the analysis of salt characteristics in combination with a literature study, KNO_3_, LiNO_3_, LiBr and LiCl stand out as promising candidates for electrolytes in REDHEs that could allow for higher power output than the commonly used NaCl and NH_4_HCO_3_. Therefore, we take a closer look at the transport properties of the respective ions through the IEMs and discuss individual ion characteristics in terms of affinity and mobility in the membrane matrix. Hydration energy, hydrated radius, and mobility of the ions determine to a large extent the interaction between ions and membrane. Their values for each cation and anion contained in the selected salts are presented in Table 5. The hydration energy affects the number of water molecules in the hydration shell of an ion and determines the affinity-based selectivity of that ion. An ion’s hydrated radius and mobility determine its size and diffusion-based transport properties inside the membrane, depending on the membrane microstructure.

The typical counter-ion exchange sequence of a common CEM containing sulfonic acid groups for monovalent cations was determined as follows [137]:

$$\begin{array}{c} K^{+} \end{array}>\begin{array}{c} {{\mathrm{NH}}_{4}}^{+} \end{array}>\begin{array}{c} {\mathrm{Na}}^{+} \end{array}>\begin{array}{c} {\mathrm{Li}}^{+} \end{array}$$

For monovalent anions, the typical counter-ion exchange sequence in a commom AEM containing quaternary ammonium groups was determined as follows [137]:

$$\begin{array}{c} {{\mathrm{NO}}_{3}}^{-} \end{array}>\begin{array}{c} {\mathrm{Br}}^{-} \end{array}>\begin{array}{c} {\mathrm{Cl}}^{-} \end{array}>\begin{array}{c} F^{-} \end{array}$$

This trend suggests that the cation permselectivity order is influenced by the Gibbs hydration energy, the hydrated size, and ion mobility. Ions with lower hydration energy can efficiently remove water molecules surrounding them. The reduced size and closer approach to the membrane’s fixed groups gives them an advantage in transferring thorough the membrane compared to ions with high hydration energy. Despite this, NH_4_^+^ having the lowest hydration energy among the studied cations comes after K^+^ in the permeation order. It has been argued that its tetrahedral shape, which is a unique feature among the studied monoatomic ions, gives ammonium a disadvantage in membrane permeation [139]. Lithium has the highest hydration energy, the biggest hydrated radius, and the lowest mobility among the cations in Table 5, and understandably comes last in the counter-ion exchange sequence for a CEM. For the AEM, the counter-ion exchange sequence follows the hydration energy of the anions, from NO_3_^−^ having the lowest hydration energy to F^−^ with the highest hydration energy. Ion mobility and hydrated radius seem to play a secondary role since Br^−^ permeates second after NO_3_^−^, despite being the smallest and having the highest mobility among the studied anions.

These trends have been confirmed for various modified membranes as well. For instance, Yang et al. modified Nafion membranes with polyelectrolyte multilayers and observed significant flux differences between K^+^ and Li^+^. The transport of Li^+^ was slower than that of K^+^ due to lower partitioning of Li^+^ ions from acidic solution into the membrane matrix and the comparatively low electrical mobility of Li^+^ ions [140]. Moreover, the higher hydration energy of Li^+^ compared to K^+^ causes Li^+^ ions to hold more water molecules around them, thereby increasing the hydrated radius when dissolved in water. This results in lower permselectivity due to size-based separation. In conclusion, based on its ionic properties, Li^+^ has disadvantages compared to K^+^ and Na^+^ when it comes to transport through the RED stack.

Sata et al. evaluated the change in transport numbers of anions including Br^−^, NO_3_^−^ and F^−^ relative to Cl^−^ ions induced by electrodialysis of the mixed solution. The selectivity order of anions through AEMs modified with different polyethylene polyamines with strongly basic anion exchange groups was the same as given above [141]. Xu et al. investigated the anion transport rate through triethyl–benzyl ammonium group modified AEMs and observed that the permselectivity order for the membrane aminated with Cl^−^ triethyl-benzyl ammonium-alcohol solution was approximately $\begin{array}{c} {\mathrm{Br}}^{-} \end{array}∼\begin{array}{c} {{\mathrm{NO}}_{3}}^{-} \end{array}>\begin{array}{c} {\mathrm{Cl}}^{-} \end{array}>\begin{array}{c} {\mathrm{Ac}}^{-} \end{array}∼\begin{array}{c} F^{-} \end{array}$ [142].

For both anions and cations, the ion permselectivity decreases with increasing hydration energy. Lower hydration energy means that the respective ion can establish a stronger bond to the fixed charged groups of the membrane than an ion with lower hydration energy. Therefore, KNO_3_ is expected to excel in terms of permselectivity among the four salts investigated more closely in this section, as K^+^ and NO_3_^−^ are the cation and anion with the lowest hydration energy in comparison. Based on the anion counter-ion exchange sequence, LiNO_3_ is the second-best suited candidate for the use in a REDHE, followed by LiBr and finally LiCl.

In a recent work, Davydov et al. have used the microheterogeneous model to determine the transport numbers of ions across IEMs for evaluating RED performance. According to the microheterogeneous model, the membrane matrix consists of two distinct phases referred to as “gel” and “electroneutral solution”. As model input parameters, a set of integral membrane properties, such as electric conductivity and diffusion permeability, was measured for both phases. As output parameter, the model calculated the transport number of ions through the membrane, which was then used to predict the open-circuit potential achievable with the respective membrane/salt system. The authors used the model to compare the performance of different membranes in RED with NaCl [46]. In addition, the model could be very useful for comparing the performance of different salts in RED with a given membrane.

In terms of non-equilibrium thermodynamics, the membrane permselectivity may be expressed by Equation (2). In this framework, the differences in permselectivities may be explained through differences in transport numbers for the various ions and membranes. For example, assuming equal salt transport numbers and identical conditions regarding the chemical potentials, a lower permselectivity would indicate a higher transport number for water, i.e., more water is transported across the membrane per mole of electrons passing in the external circuit. However, Equation (2) also highlights that membrane permselectivities for different salts must be compared for equivalent conditions in terms of the chemical potentials rather than equal salt concentrations. Therefore, further studies are required to give insight into and compare the permeabilities of the selected salts in commercial IEMs and evaluate the potential of tailor-making IEMs to increase the permselectivity for the respective salts.

## 6. Conclusions

The present work gives a comprehensive overview of the reverse electrodialysis heat engine (REDHE) concept, where a reverse electrodialysis stack as a power unit is coupled in a closed loop with a thermal regeneration unit to restore the salinity gradient. Benefits offered by this technology include

- possibility for green hydrogen production,
- free choice of electrolyte due to closed-loop operation,
- free choice of solvent,
- mitigation of membrane fouling,
- no pre-treatment required,
- possible use of low-grade waste heat for solution regeneration.

Despite these opportunities, conversion efficiencies and profits yielded with REDHE have not yet met industrial demands. Table 6 summarizes key parameters together with the determining process and material properties for both the power and the regeneration unit. One of the main challenges in the optimization of REDHE is to increase the power density of the unit which can be achieved both trough membrane development and through process design. For example, high-performance polymers featuring a low area resistance while maintaining sufficient permselectivity are a main target in membrane development. Profiled membranes have been developed with the purpose to replace spacers, improving the hydrodynamic flow and reducing the pressure drop across the RED stack. Spacerless design allows for reduced intermembrane distances, which decreases the Ohmic resistance and therefore allows for increased gross power density. The concentration of the low-concentrated compartment is limited by the trade-off between maximizing the chemical potential across the membrane and minimizing the Ohmic resistance. Multi-step RED can contribute to increased power density while controlling the Ohmic resistance. The use of salts other than sodium chloride and ammonium bicarbonate, which may have superior characteristics for the use in REDHE, has just recently gained academic attention. In the RED unit, the permselectivity is the figure of merit indicating the suitability of a salt. However, salt properties are even more decisive for the efficiency of the regeneration unit, where vast amounts of energy are needed to restore the initial concentration difference between the high-concentrated and the low-concentrated stream after they passed through the RED stack. Use of highly soluble or thermolytic salts, use of organic solvents, and multi-step regeneration units are suggested for improving the specific thermal consumption. While, in general, the hydrogen output rate is higher with evaporation, precipitation requires less heat input for the electrolyte regeneration. The temperature dependency of the solubility for specific salts like KNO_3_, NH_4_HCO_3_, and RbNO_3_, suggests that a significantly higher concentration difference between the high-concentration and low-concentration streams can be obtained by increasing the upper temperature (e.g., to 80 °C). This is especially relevant when using precipitation as a regeneration unit. Due to relatively high conductivity, high solubility temperature dependency, and beneficial enthalpy of mixing, salts such as KF, LiNO_3_, LiBr and LiCl have the potential to be high performers in terms of hydrogen production for both of the regeneration processes. In terms of permeability through ion-exchange membranes, KNO_3_ features excelling ionic characteristics.

The systematic review presented in this article offers insights that inform REDHE development regarding preferable salts, ion-exchange membrane development, and solution regeneration. The authors suggest that the next steps towards industrialization for REDHE is to develop a comprehensive thermodynamic model to understand the most decisive parameters for the choice of salt and regeneration unit, experimentally validate the results and informed by this, design high-performance ion-exchange membranes tailored to the respective salts. Furthermore, to facilitate the design of highly efficient REDHEs, future studies should investigate how the relevant salts perform in the power unit and the regeneration step.

## Figures and Tables

**Table 2:** Studies on RED using commercial membranes.

Application	$E_{\mathrm{OCV}}$	$R_{\mathrm{stack}}$	$P_{d}$	Rinse Solution	α	T	Membrane	$d_{m}$	$d_{s}$	$V_{f}$	Ref.
	(V)	(Ω)	($W\;m^{-2}$)			(${}^{\circ }$C)		(μm)	(μm)	($L\cdot h^{-1}$)	
NaCl, 25 cell pairs, 10 × 10 cm	3.48–4.10	12.8–32.4	0.38–1.41	0.3 M C_6_FeK_4_N_6_ 0.3 M C_6_FeK_3_N_6_ 2.5 M NaCl	0.68	10–60	Fuji-AEM-80045 Fuji-CEM-80050	129 114	270	20–40	[75,76]
1.5 M/0.02 M NH_4_HCO_3_ 20 cell pairs, 10.5 × 7.5 cm	3.07	25	0.33	0.1 M C_6_FeK_4_N_6_ 0.1 M C_6_FeK_3_N_6_	0.88	amb.	Selemion AMV Selemion CMV	130	500	48	[57]
(a) 0.14 M/3.6 M NaCl (b) 0.0015 M/1.5 M NH_4_HCO_3_ 10 cell pairs 8 × 8 cm	(a) 1.08 (b) -	(a) 0.62 (b) 0.32	-	0.6 M NaCl	-	amb.	PCCell PC-SA PCCell PC-SK	-	500	0.6 (HC) 1.2 (LC)	[54]
(a) 0.66 M/0.0036 M NaCl (b) 5 M/0.1 M NaCl (c) 5 M/1 M NaCl 20 cell pairs 6.3 × 32 cm	(a) 4.11 (b) 2.63 (c) 0.88	(a) - (b) 2.0 (c) 0.25	(a) 0.5 (b) - (c) -	0.05 M C_6_FeK_4_N_6_ 0.05 M C_6_FeK_3_N_6_ 0.25 M NaCl	(a) - (b) 0.68 (c) 0.46	24	Fumatech FAS-50 Fumatech FKS-50	50	270	12	[77]
brine/brackish water 125 cell pairs 44 × 44 cm	15.4	1.2	1.6	0.3 M FeCl_2_ 0.3 M FeCl_3_ 2.5 M NaCl	-	26	Fujifilm: AEM 80045-01 CEM 80045-04	120	280	480	[21]
brine/brackish water 10 cell pairs 8 × 11 cm	2.1	4.5	0.5	3 M NaCl	-	20	Neosepta AMX Neosepta CMX	-	200	-	[78]
0.02 M/0.5 M NaCl 50 cell pairs 10 × 10 cm	-	17	0.93	0.05 M C_6_FeK_4_N_6_ 0.05 M C_6_FeK_3_N_6_ 1 M NaCl	-	25	Fumasep FAD Fumasep FKD	82	200	42	[79]
0.01/5 M NaCl 5 cell pairs 10 × 10 cm	-	-	3.8	0.1 M C_6_FeK_4_N_6_ 0.1 M C_6_FeK_3_N_6_ 0.5 M NaCl	-	25	Neosepta ACS Neosepta CMS	-	100	1.5	[80]
0.507 M/0.017 M NaCl 5 cell pairs 10 × 10 cm	-	-	≤ 2.2	0.025 M C_6_FeK_4_N_6_ 0.025 M C_6_FeK_3_N_6_ 0.25 M NaCl	-	25	Fumatech FAS Fumatech FKS	30–40	60–485	0.06–15	[81]
5 M/0.05 M NaCl 1 cell pair 13 × 9 cm	(a) 0.115 (b) 0.118	-	(a) 1.5 (b) 2.0	0.5 M FeCl_2_ 0.5 M FeCl_3_ 1.0 M NaCl	0.5–0.7 0.7–0.8	(a) 25 (b) 40	Fumatech FAS-50 Fumatech FKS-50	50	155	0.42	[15]

*E_OCV_*—open circuit potential, *R*_stack_—stack resistance, *P_d_*—peak power density, *a*—permselectivity, T—temperature, *d_m_*—membrane thickness, *d_s_*—spacer thickness, *V_f_*—flow rate of HC and LC streams. First column: type of salt and concentration of HC and LC steam when available, number of cell pairs, and single membrane area.

**Table 4:** Potential electrolytes for the use in REDHE with saturation concentrations in water at T=10 °C, T=40 °C, and T=80 °C, and molar enthalpies of solution at infinite dilution (assumed constant in temperature range) [132].

Compound	Formula	$M_{i}$ (g/mol)	Aqueous Solubility $m^{\mathrm{sat}}$ (mol/kg)			${Τ}_{\mathrm{sol}}H$ (kJ/mol)
			at 10 ${}^{\circ }$C	at 40 ${}^{\circ }$C	at 80 ${}^{\circ }$C	
Ammonium Bromide	NH_4_Br	97.94	6.86	9.16	12.28	16.78
Ammonium Chloride	NH_4_Cl	53.49	6.27	8.58	12.15	14.78
Ammonium Bicarbonate	NH_4_HCO_3_	79.06	2.01	4.89	17.54	26.09
Lithium Bromide	LiBr	86.85	17.34	24.25	27.79	−48.83
Lithium Chloride	LiCl	42.39	17.41	21.17	26.58	−37.03
Lithium Nitrate	LiNO_3_	68.95	8.74	21.85	29.71	−2.51
Potassium Bromide	KBr	119.0	5.00	6.39	8.01	19.87
Potassium Chloride	KCl	74.55	4.15	5.37	6.87	17.22
Potassium Fluoride	KF	58.10	11.38	24.55	25.82	−17.73
Potassium Nitrate	KNO_3_	101.1	2.11	6.22	16.84	34.89
Sodium Bromide	NaBr	102.9	8.25	10.36	11.64	−0.60
Sodium Chloride	NaCl	58.44	6.11	6.22	6.49	3.88

**Table 5:** Hydrated radii [135], hydration energy [136] and mobility in the water [137] of selected anions and cations.

Ion	Hydrated Radius (nm)	Hydration Energy (kJ/mol)	Mobility in Water ($10^{-8}\;m^{2}/\mathrm{sV}$)
Na^+^	0.358	−365	5.19
Li^+^	0.382	−475	4.01
K^+^	0.331	−295	7.19
NH_4_^+^	0.331	−285	7.63 ${}^{b}$
Cl^−^	0.332	−340	7.91
F^−^	0.352	−465	5.70
NO_3_^−^	0.335	−300	7.40
Br^−^	0.330	−315	8.09
HCO_3_^−^	0.439 ${}^{a}$	−335	-

*^a^* value extracted from [138], *^b^* value extracted from [102].

**Table 6:** Overview of the key parameters and characteristics for the REDHE stack.

Component		Key Parameters	Determined by
RED stack	Membrane properties	Permselectivity and electrical resistance	- ion-exchange capacity - water uptake - fixed charge density
	Ion Characeristics	Affinity and mobility, open circuit potential	- hydration energy - hydrated radius - conductivity of solution - chemical potential of salt and water - activity coefficient ratio
	Hydrodynamic Design	hydrodynamic losses/ pressure drop	- flow channel dimensions - spacer selection - manifolding system - dead spots in flow channels
Regeneration unit	Evaporation - higher H_2_ output - less membrane area required Precipitation - less heat required	Restored salinity gradient, heat requirement	- salt solubility - temperature dependency of solubility - dissolution enthalpy change
